# Translational Experimental Basis of Indirect Adenosine Receptor Agonist Stimulation for Bone Regeneration: A Review

**DOI:** 10.3390/ijms25116104

**Published:** 2024-06-01

**Authors:** Quinn T. Ehlen, Nicholas A. Mirsky, Blaire V. Slavin, Marcelo Parra, Vasudev Vivekanand Nayak, Bruce Cronstein, Lukasz Witek, Paulo G. Coelho

**Affiliations:** 1 University of Miami Miller School of Medicine, Miami, FL 33136, USA; 2 Center of Excellence in Morphological and Surgical Studies (CEMyQ), Faculty of Medicine, Universidad de la Frontera, Temuco 4811230, Chile; 3 Department of Comprehensive Adult Dentistry, Faculty of Dentistry, Universidad de la Frontera, Temuco 4811230, Chile; 4 Department of Biochemistry and Molecular Biology, University of Miami Miller School of Medicine, Miami, FL 33136, USA; 5 Department of Medicine, New York University Grossman School of Medicine, New York, NY 10016, USA; 6 Biomaterials Division, NYU Dentistry, New York, NY 10010, USA; 7 Department of Biomedical Engineering, New York University Tandon School of Engineering, Brooklyn, NY 11201, USA; 8 Hansjörg Wyss Department of Plastic Surgery, New York University Grossman School of Medicine, New York, NY 10016, USA; 9 DeWitt Daughtry Family Department of Surgery, Division of Plastic Surgery, University of Miami Miller School of Medicine, Miami, FL 33136, USA

**Keywords:** adenosine, indirect agonist, bone regeneration, dipyridamole

## Abstract

Bone regeneration remains a significant clinical challenge, often necessitating surgical approaches when healing bone defects and fracture nonunions. Within this context, the modulation of adenosine signaling pathways has emerged as a promising therapeutic option, encouraging osteoblast activation and tempering osteoclast differentiation. A literature review of the PubMed database with relevant keywords was conducted. The search criteria involved in vitro or in vivo models, with clear methodological descriptions. Only studies that included the use of indirect adenosine agonists, looking at the effects of bone regeneration, were considered relevant according to the eligibility criteria. A total of 29 articles were identified which met the inclusion and exclusion criteria, and they were reviewed to highlight the preclinical translation of adenosine agonists. While preclinical studies demonstrate the therapeutic potential of adenosine signaling in bone regeneration, its clinical application remains unrealized, underscoring the need for further clinical trials. To date, only large, preclinical animal models using indirect adenosine agonists have been successful in stimulating bone regeneration. The adenosine receptors (A_1_, A_2A_, A_2B_, and A_3_) stimulate various pathways, inducing different cellular responses. Specifically, indirect adenosine agonists act to increase the extracellular concentration of adenosine, subsequently agonizing the respective adenosine receptors. The agonism of each receptor is dependent on its expression on the cell surface, the extracellular concentration of adenosine, and its affinity for adenosine. This comprehensive review analyzed the multitude of indirect agonists currently being studied preclinically for bone regeneration, discussing the mechanisms of each agonist, their cellular responses in vitro, and their effects on bone formation in vivo.

## 1. Introduction

The management of critical-sized bone defects remains a major challenge to health care providers as well as patients. Critical-sized bone defects, defined as those that will not heal spontaneously, requiring surgical intervention, occur at an incidence of approximately 100,000 cases annually in the United States and result in an estimated healthcare cost of ~$2.5 billion dollars [1,2,3]. A variety of underlying diseases and conditions (such as osteoporosis, periodontal disease, congenital alveolar cleft defects, and bone tumors) may further exacerbate the bone healing process. Additionally, risk factors such as aging, alcohol, tobacco, and steroid use may contribute towards increasingly delayed and/or impaired bone healing [4].

The current standard of care for reconstruction of defects involves surgical intervention utilizing autogenous bone grafts (autografts) due to their established osteoconductive, osteogenic, and osteoinductive properties [5]. This type of grafting has been used in a wide range of procedures, including alveolar distraction, dental implantation, and various head and neck procedures [6,7,8]. Autografts are harvested from various bones; however, the fibula is a common donor site due to its non-weight-bearing status [6]. However, this type of bone tissue transfer is not without its respective drawbacks, such as donor site morbidity, muscle weakness, potential for infection, and pain [9,10]. Further, autografts may lack structural support during the healing process due to poor stabilization and limited bone contact, which can lead to weakness of the reconstructed segment [6,11]. Despite their popularity, autografts may fail to incorporate in up to 60% of cases after 10 years of surgical reconstruction [12]. Such limitations highlight the need for alternative strategies in bone regeneration.

**Figure 1:**
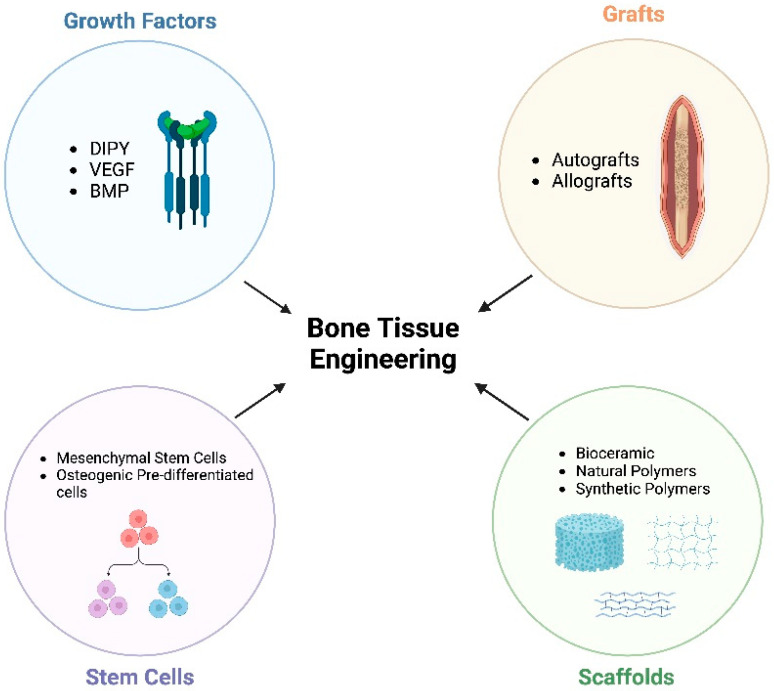
Components of bone tissue engineering (created with Biorender.com). Dipyridamole (DIPY), vascular endothelial growth factor (VEGF), bone morphogenic protein (BMP).

A variety of bone tissue engineering (BTE) strategies (Figure 1) have been under investigation to ameliorate such drawbacks. BTE is a rapidly evolving field that seeks to integrate the principles of biology and engineering to develop materials and methodologies capable of replacing or regenerating damaged bone tissue. Fundamental to this field is the development of tissue engineering devices that support the mechanical structure of bone while simultaneously promoting the biological processes necessary for bone repair and growth [13]. Tissue engineering devices, such as scaffolds, meticulously designed for custom fit-and-fill bony defect repair, are often enhanced with components such as stem cells and bioactive molecules to optimize the healing process [14]. These additives seek to improve the bone regeneration capabilities of tissue engineering devices such as scaffolds while avoiding the shortcomings associated with autografts. The integration of stem cells and bioactive molecules into the scaffolds has demonstrated an increased rate and quantity of bone formation when compared to scaffolds without these additives [15,16]. A well-studied and validated bioactive molecule for bone regeneration is recombinant human bone morphogenetic protein 2 (rhBMP-2) [14,17]. This growth factor is involved in committing multipotent stromal cells toward an osteogenic lineage for the formation of new bone [18]. While rhBMP-2 is currently approved for use in treatment of long bone fractures, clinical studies have demonstrated critical side effects, including vertebral osteolysis, ectopic bone formation, radiculitis, and stimulation of cancer growth [19,20,21]. Vertebral osteolysis is the breakdown of vertebrae, leading to spinal instability, which has been described when using rhBMP-2 in lumbar fusion procedures [22]. Ectopic bone formation refers to the development of bone in non-native locations throughout the body [23]. Similarly, excessive bone formation has been reported to cause radiculitis, or inflammation of the nerve roots exiting the spinal column [24]. Because of these potential complications, investigators are actively investigating alternative bioactive molecules, such as adenosine receptor agonists, as additives to scaffolds to facilitate bone regeneration.

Adenosine, an extracellular purine generated by all cells from the hydrolysis of adenine nucleotides, has been recognized for its physiological functions through the activation of cell-surface receptors for over a century [25]. Its uses are vast, producing different effects on various organs. For example, its first effect historically was found in cardiac muscle, resulting in decreased heart rate and vasodilation of the coronary arteries [26]. Through its vasodilatory effect, it has been shown to decrease ischemia to organs such as the liver, kidney, heart, skin, and lung [27,28,29,30]. Adenosine has also demonstrated an effect on immune cells and has been useful in inflammatory diseases such as asthma, chronic obstructive pulmonary disease (COPD), inflammatory bowel disease, and arthritis [31,32,33]. Additionally, adenosine has been shown to be essential in wound healing, promoting granulation tissue and extracellular matrix production [34,35]. Investigators are beginning to understand its effects on bone tissue formation, which has emerged as a key metabolic pathway that can contribute to various phases of bone hemostasis and regeneration [36]. The goal of this review is to provide a comprehensive evaluation of adenosine’s role in bone regeneration, as well as present the benefits and current applications of indirect adenosine agonists within in vivo and in vitro studies. Additionally, this work aims to highlight future directions of indirect adenosine agonists in clinical applications as well as their limitations.

## 2. Methods

**Figure 2:**
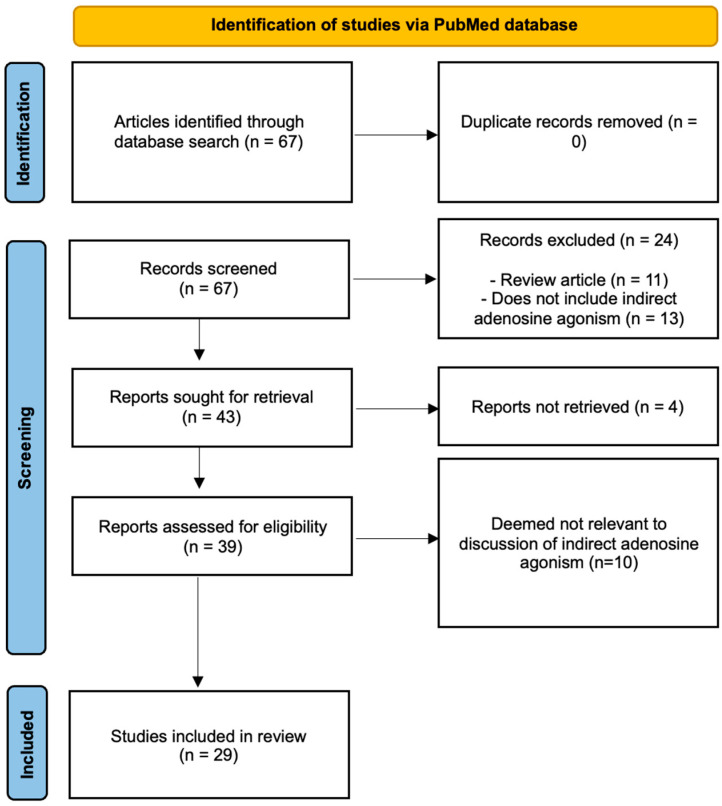
Preferred Reporting Items for Systematic Reviews and Meta-Analysis (PRISMA) flow diagram of the literature search.

A literature review of the PubMed database with relevant keywords—adenosine agonism, bone regeneration, dipyridamole, ticagerlor, Equilibrative Nucleoside Transporter-1 (ENT-1) inhibitors, CD39/73 agonist, adenosine deaminase, and adenosine kinase—was used to compile the tables. Key terms, Medical Subject Headings (MeSH) terms, and Boolean operators (‘AND’ and ‘OR’) were used across each database to refine our search. The search strategy was collectively reviewed by members of the review team prior to execution using the Peer Review of Electronic Search Strategies (PRESS) checklist. Studies were included if they involved in vitro or in vivo models, with clear methodological descriptions. Only studies that included the use of indirect adenosine agonists, looking at the effects of bone regeneration in preclinical/clinical studies, were considered relevant. Careful consideration was given to article references to ensure all relevant studies were included. Exclusion criteria were non-peer-reviewed articles, case reports, commentaries, editorials, and reviews without original data. Although the number of studies involving indirect adenosine agonism on bone regeneration are plentiful in the literature, the 29 articles referenced and summarized in both tables are representative in nature to highlight preclinical translation of adenosine agonists in multiple models within recent years (Figure 2).

## 3. Results

### 3.1. Adenosine’s Role in Bone Regeneration

Bone turnover takes place at discrete sites and is regulated at a local level by paracrine/autocrine signaling. In addition to well-documented effects on various organs, adenosine has shown to play a key role in this extracellular signaling process, modulating the environment to enable proper bony regeneration [37]. Under ordinary physiological conditions, adenosine is found in low concentrations and has an extremely short half-life of <10 s [38]. However, during periods of physiological and cellular stress, such as during bone loading, bone fracture, and bone repair, adenosine concentrations increase [37,39]. Though its effect is dependent on its concentration, target cell composition, and the density of the respective adenosine receptors on the cell membrane, adenosine can create an immunological response that is either pro-inflammatory or anti-inflammatory through agonism of adenosine receptors [40,41,42]. Agonism of adenosine receptors can stimulate cells to upregulate or downregulate various cytokines. Adenosine also serves as a chemotaxis agent, attracting neutrophils and other immunologic cells. In addition to its immunologic role, adenosine acts to stimulate extracellular matrix synthesis by increasing collagen production through the mitogen-activated protein (MAP) kinase pathway [43,44]. Additionally, adenosine has been shown to increase calcium mobilization extracellularly and promote angiogenesis via the increased production of vascular endothelial growth factor (VEGF) [45]. Through these mechanisms, the adenosine receptors and their respective pathways can stimulate the cellular pathways necessary for bone regeneration.

### 3.2. Function of Adenosine Receptors

**Figure 3:**
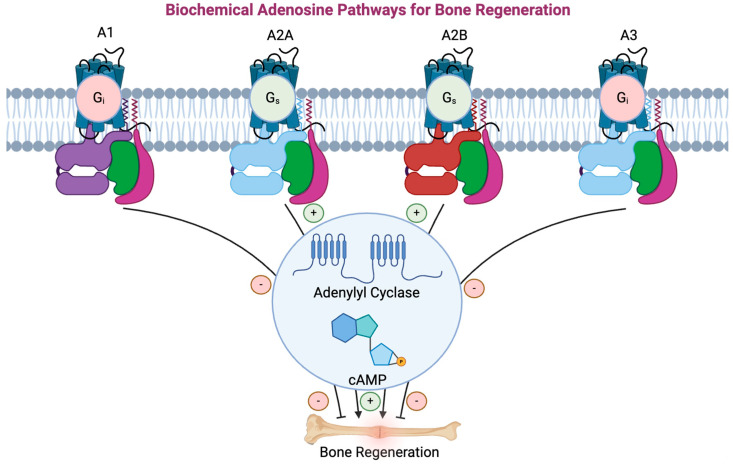
Biochemical pathways of adenosine signaling with respective adenosine receptors and their effects on bone regeneration. A_2A_ and A_2B_ produce anti-inflammatory responses, whereas A_1_ and A_3_ produce pro-inflammatory responses (Created with BioRender.com).

It has been discovered that adenosine has various receptors present on different cells that stimulate various intracellular pathways. There are four adenosine receptor subtypes: A_1_, A_2A_, A_2B_, and A_3_ (Figure 3), all of which are G proteins that consist of a single polypeptide chain which crosses the cell membrane seven times [46]. The A_1_ receptor is classified as a G_i_ protein: this receptor inhibits adenylyl cyclase, protein kinase C (PKC), MAP kinase, phosphoinositide 3-kinase (PI3), and calcium channels [46]. This causes decreased intracellular cyclic adenosine monophosphate (cAMP). Additionally, activation of the A_1_ receptor activates potassium channels [47]. Previous studies have reported that although A_1_ was expressed in osteoblast precursors, it was involved in the induction of adipocyte differentiation rather than osteoblast differentiation [17,48].

The A_2A_ receptor is a high-affinity adenosine receptor, classified as a G_s_ protein [47]. This receptor stimulates adenylyl cyclase, activates protein kinase A (PKA) and MAP kinase, and phosphorylates cAMP-response element binding protein (CREB) [49,50,51]. CREB is a transcription factor that can influence gene expression. Mediero et al. found that ligation of the A_2A_ receptor inhibited osteoclast formation, theoretically preventing the breakdown of bone [52]. The group also demonstrated that an A_2A_ inhibited osteoclast differentiation in a reversible manner, in addition to decreasing the inflammatory response, as seen with decreased Interleukin-1B (IL-1B) and Tumor Necrosis Factor alpha (TNF-α) secretion [52].

The A_2B_ receptor is a low-affinity adenosine receptor with both G_s_ and G_q_ activity [47]. Due to its low affinity, a very high extracellular concentration of adenosine is required to activate these receptors. The A_2B_ receptor activates phospholipase C (PLC), subsequently activating adenylyl cyclase and increasing the intracellular concentration of cAMP and calcium [53]. Agonism of the A_2A_ and A_2B_ receptors has been shown to stimulate angiogenesis and endothelial cell proliferation, aiding wound healing [34].

Finally, the A_3_ receptor, also classified as a G_i_ protein, is commonly overexpressed in inflammatory and neoplastic cells [54]. This receptor functions similarly to A_1_, inhibiting adenylyl cyclase to decrease intracellular cAMP and downregulate the inflammatory nuclear factor kappa B (NFκB) pathway [53,54]. However, no direct effects of A_3_ stimulation or blockade have been reported in either osteoclastogenesis or osteoblast bone production [55]. Nonetheless, the binding of adenosine to these respective receptors is dependent on the extracellular concentration of adenosine as well as the density of each receptor at the cell surface.

**Figure 4:**
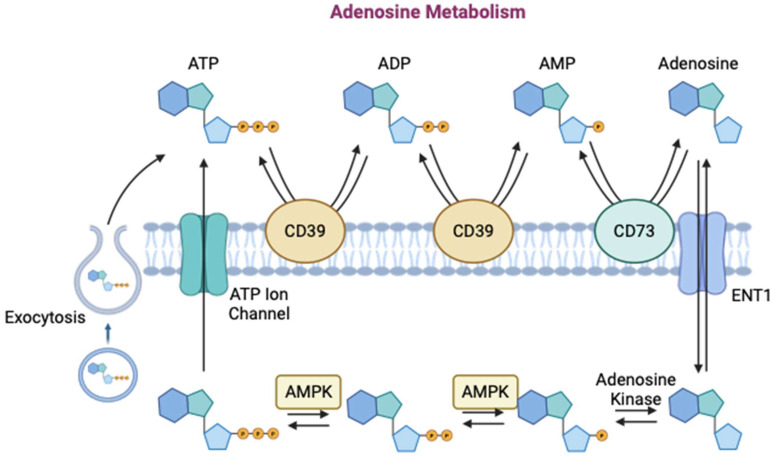
Schematic depicting adenosine metabolism and the enzymes affecting the extracellular concentration of adenosine (created with BioRender.com).

The extracellular concentration of adenosine, a byproduct of cellular metabolism, is a function of the activity of various enzymes in the body (Figure 4). Extracellularly, CD39 first hydrolyzes adenosine triphosphate (ATP) to adenosine diphosphate (ADP), and then once more to form adenosine monophosphate (AMP) [53]. CD73 then dephosphorylates AMP to form adenosine in its active form [53]. Intracellularly, ATP and S-adenosyl homocysteine are broken down by ATPase to form adenosine. The extracellular concentration of adenosine is therefore a function of the activity of these enzymes as well as the release or uptake of ATP and adenosine by various cells [56]. ATP can be released from cells through opening of ATP-permeable ion channels, exocytosis of vesicles with ATP, or exocytosis of ATP-permeable ion channel-containing vesicles. Adenosine is transported across the cell membrane by equilibrating nucleoside transporters (ENTs) [56]. Therefore, cellular metabolism, biochemical gradients, and reaction kinetics all play a role in determining the extracellular concentration of adenosine.

**Figure 5:**
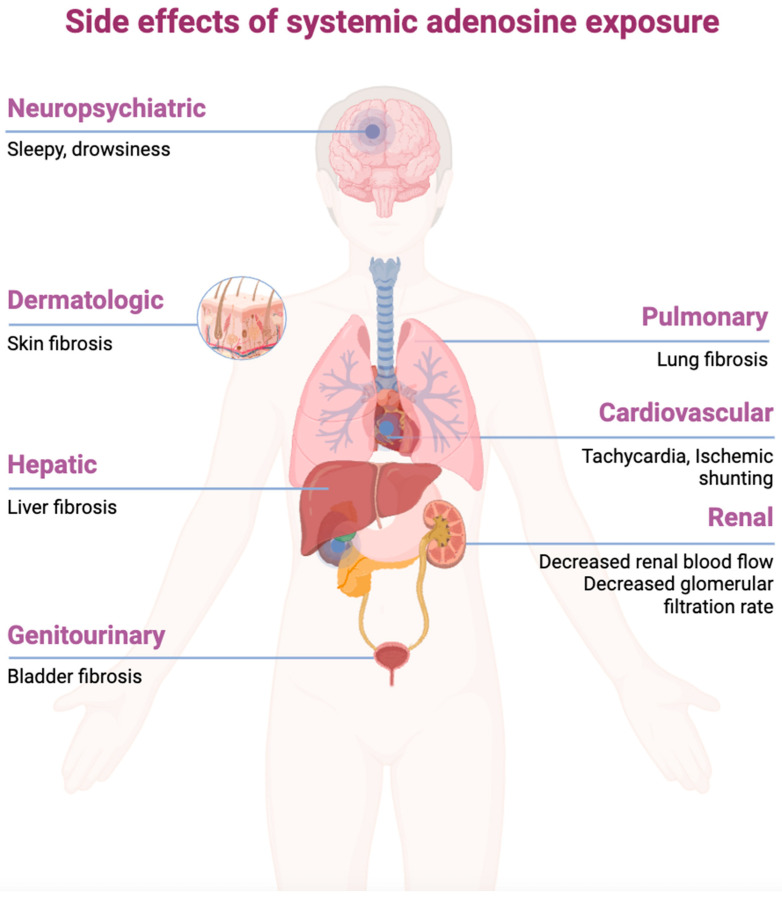
Potential side effects of the systemic administration of adenosine (created with BioRender.com).

While adenosine itself is efficacious in stimulating biochemical processes in many organ systems, its therapeutic value is limited by its side effects when administered systematically. Because of the widespread nature of adenosine signaling, agonism of the various receptors can cause pathophysiological dysfunction (Figure 5). Activation of the A_1_ receptor has been shown to decrease renal blood flow and the glomerular filtration rate [57]. Activation of the A_1_ and A_2A_ receptors is speculated to promote sleep, leading to drowsiness [57]. Activation of the A_2B_ receptor has been shown to promote tumor growth in the bladder and breast [58]. The pro-inflammatory aspect of adenosine signaling indicates that chronic agonism can lead to tissue injury and fibrosis of the lungs, liver, skin, and penis [59]. Another downfall of systematic adenosine use is its extremely short half-life, between 1 and 10 s [60]. As adenosine receptors are found globally, and adenosine is pivotal in cellular metabolism, the molecule is briskly cleared from plasma by cell transport and enzymatic degradation [60]. This problem has been addressed by novel drug delivery mechanisms, including core–shell nanofibers, which can release adenosine gradually over a longer duration [61]. However, some of the other side effects of systematic adenosine administration may persist. 

### 3.3. Indirect Agonists

To avoid the side effects associated with direct adenosine agonists, indirect agonists have been developed to increase endogenous levels of adenosine locally. This has the potential to provide tissue-specific stimulation, thereby avoiding systematic side effects [57]. Indirect agonists act on the enzymes and transporters that affect the extracellular concentration of adenosine, either by stimulating adenosine synthesis, by inhibiting adenosine degradation, or by changing its transport across the cell membrane [57]. These compounds can be used to increase the extracellular concentration of adenosine and agonize the adenosine receptors. Current indirect adenosine agonists being studied in the use of bone regeneration include dipyridamole (DIPY), ticagrelor, CD39/73 agonists, adenosine deaminase inhibitors, ENT-1 inhibitors, and adenosine kinase inhibitors. Table 1 and Table 2 provide a list of such in vivo and in vitro studies examining the effects of indirect adenosine agonism on bone regeneration in the last 25 years.

#### 3.3.1. DIPY

DIPY, an indirect adenosine agonist, is an extensively studied biologic compound [55,62,63,64,65,65,66,67,67,68,68,69,81,82,83,84,85,86,87,88,89,90,91,92,93,94,95,96,97]. Historically, it has been used as a vasodilatory and antiplatelet agent [98,99,100,101]. It acts to increase extracellular adenosine by inhibiting ENT-1 and preventing the degradation of adenosine [102]. Specifically, DIPY administration induces agonism of the A_2A_ receptor, which increases intracellular cAMP and stimulates extracellular matrix formation [49,65]. Stimulation of the various adenosine receptors has other effects, as mentioned previously. These biochemical pathways have been shown to stimulate osteoblast differentiation and proliferation, which can aid in bone regeneration.

In vitro studies have suggested that DIPY has osteoinductive potential. Mediero et al. studied bone marrow cells from mouse femurs and analyzed osteoclast differentiation and osteogenesis in the presence of DIPY [69]. The group observed that DIPY markedly inhibited osteoclast differentiation and, while not increasing osteoblast production, increased the synthesis of osteogenic proteins from osteoblasts [69]. To determine the receptor to which this effect can be attributed, they administered a direct A_2A_ agonist, CGS21680 [17]. This agonist was determined to produce the same effect of decreased osteoclast differentiation and increased osteoblast markers. It is speculated that DIPY-induced bone regeneration primarily acts through the A_2A_ receptor [17]. The direct agonist, CGS21680, was found to have a dose-dependent effect on inhibiting osteoclastogenesis, but can be reversed by an A_2A_ antagonist [52]. This finding confirms that adenosine’s effect on osteogenesis can be produced by endogenous adenosine and acts in an autocrine manner [52]. A separate study analyzed the effect of tenofovir, an HIV antiviral, on bone catabolism [103]. As an AMP analog, it was postulated that tenofovir can cause decreased mineral bone density and increased bone catabolism. This effect was confirmed in the study with the inhibition of osteoblast activity and differentiation [103]. Interestingly, it was determined that DIPY could counteract this effect by inhibiting osteoclasts and stimulating osteoblasts, thereby minimizing the bone loss induced by tenofovir. The suspected mechanism included increased extracellular adenosine concentration, stimulating the anabolic purinergic biochemical pathways [103].

**Figure 6:**
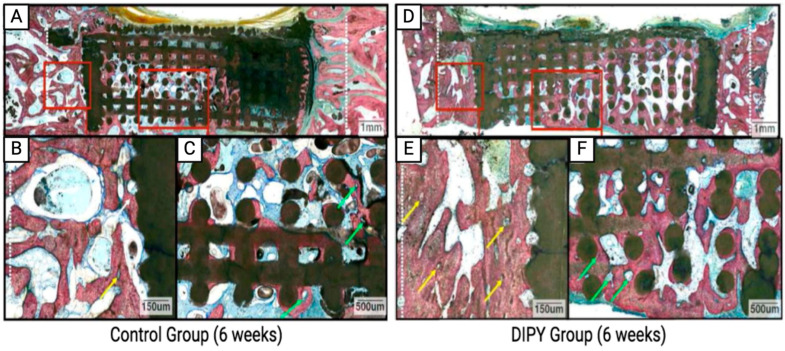
Representative histologic comparison from calvarial defect model treated with naked scaffold (**A**–**C**) versus DIPY-treated scaffold (**D**–**F**). Red boxes in (**A**,**D**) indicate magnified images of (**B**,**C**) and (**E**,**F**), respectively. Green arrows indicate osteon development and angiogenesis. White arrows indicate bone growth between the defect margin (dashed white lines) and scaffold wall. Yellow arrows highlight the primary osteons. Reproduced with permission from [82].

Initial studies conducted by Mediero et al. of A_2A_ agonism on bone regeneration were further supported by a series of in vivo studies performed using a mouse model, which demonstrated that A_2A_ knockout mice had lower bone density and increased osteoclast activity [52]. Ishack et al. further studied the effect of an A_2A_ agonist on implant integration in a mouse calvarium model, finding that bone resorption markers were diminished in the treatment groups. Other studies have confirmed that A_2A_ agonism can increase bone, decrease osteoclasts, and enhance implant survival [104]. These findings of A_2A_ agonism inspired the use of DIPY as a potential treatment for bone regeneration. In a rabbit alveolar model, 3D-printed bioceramic scaffolds were coated with either DIPY, rhBMP-2, or control (uncoated) scaffolds, then implanted into critical-sized defects [17]. While the control scaffolds did not elicit healing, the DIPY and rhBMP-2 groups regenerated bone similarly. However, scaffolds coated in rhBMP-2 showed signs of osteolysis and early craniofacial suture fusion, while DIPY-coated scaffolds promoted uneventful or normal healing [64]. To understand the mechanism in vivo, A_2A_ knockout mice with the same defect were treated with DIPY, resulting in minimal bone growth, similar to the control groups [17]. This confirmed that DIPY-induced bone regeneration occurs primarily via the A_2A_ receptor. In a similar rabbit craniofacial model, 3D-printed bioceramic scaffolds were uncoated (control) or coated with DIPY. It was demonstrated that scaffolds augmented with DIPY generated vascularized bone comparable to autogenous bone graft and native bone with no adverse effects, such as premature cranial suture fusion or asymmetrical craniofacial growth [65]. These results were confirmed in a separate, critically sized mice calvarium defect model in which DIPY-treated scaffolds had increased osteoblast activity with decreased osteoclasts, leading to increased bone regeneration (Figure 6) [17]. Similar results were reproduced in a rabbit mandible and calvarium model, which emphasized that DIPY administration did not produce an inflammatory response different from the control [66,67]. While the immune response mechanism of adenosine signaling in bone regeneration is less studied, it is known that the adenosine receptors are present on macrophages and other immune cells and contribute to pro-inflammatory and anti-inflammatory responses [105]. Additionally, while there has previously been concern that adenosine signaling using DIPY may produce a pro-inflammatory response, leading to fibrosis and malformation, A_2A_ receptor stimulation has been shown to produce an anti-inflammatory response [82] without ectopic bone formation [67,68].

Continuing with pre-clinical research, particularly with the use of larger translational models, DeMitchell-Rodriguez et al. used a critical-sized porcine calvarium model to study DIPY in bone regeneration [63]. The treatment group produced more bone than the negative controls with native mechanical properties, an absence of ectopic bone formation, and excessive inflammatory response [63]. Other large animal models have studied different devices in different anatomical locations. Where, Pacheco-Vergara et al. investigated the impact of DIPY on the osteointegration of titanium implants in sheep vertebrae [62]. It was found that DIPY-treated animals showed further bone integration and regeneration at 3 weeks post-operation. No differences were seen at 6 or 12 weeks post-operation, indicating that DIPY may play a beneficial role in the early stages of osteointegration [62]. While there have been extensive pre-clinical models, DIPY has yet to be tested clinically. Further large animal models are necessary to bridge the gap for subsequent testing in humans.

#### 3.3.2. Ticagrelor

Ticagrelor primarily serves as P2Y_12_ inhibitor to prevent platelet aggregation and thrombosis [106]. However, it also acts through a similar mechanism to DIPY by inhibiting the ENT-1 protein on the cell membrane. This increases the extracellular concentration of adenosine, thereby increasing ligation on adenosine receptors. Similar to DIPY, it is hypothesized that this inhibition of ENT-1 can be harnessed to indirectly stimulate A_2A_ agonism and promote bone regeneration by activating osteoblasts, inhibiting osteoclast differentiation, and increasing extracellular matrix production [49,65]. Another mechanism by which ticagrelor may affect bone regeneration is its effect on vitamin D. Clinically, it was found that vitamin D levels can alter the platelet reactivity in patients undergoing treatment with ticagrelor. It is suspected that the efficacy of ticagrelor is dependent on vitamin D levels and, as such, its administration may lower vitamin D [107]. However, it is uncertain how this phenomenon affects bone regeneration and further discovery is required to uncover the exact mechanisms [107].

Initial in vitro studies of ticagrelor were conducted to determine its effects on adenosine signaling and bone regeneration. Using murine bone marrow-derived precursors, it was determined that ticagrelor inhibited osteoclastogenesis through inhibition of ENT-1 [69]. However, it was deemed to be less potent than DIPY [69]. Through the administration of various adenosine receptor antagonists, it was established that ticagrelor acted through an A_2A_ mechanism, consistent with DIPY [69]. Another study examined the effect of ticagrelor on the osteogenic differentiation of MSCs [80]. The ticagrelor group showed significant increases in extracellular matrix production, alkaline phosphatase activity, and increased bone markers while displaying decreased IL-6 and TNF-α expression [80]. This osteogenic differentiation aligns with many other studies that support adenosine signaling in bone regeneration [69].

Additionally, several preclinical studies using animal models have been conducted to study the efficacy of ticagrelor in vivo. In a critical-sized mouse calvarium model, scaffolds treated with ticagrelor resulted in greater bone regeneration than control scaffolds [69]. The experimental group demonstrated increased bone volume and bone mineral density [69]. The ticagrelor treatment performed similarly to scaffolds treated with BMP-2. The mechanism was determined to be A_2A_ agonism, as the ticagrelor effect was abrogated in A_2A_ knockout mice [69]. Instead of local delivery, Kobat et al. studied the systemic administration of ticagrelor in bone regeneration [70]. The tibial mice model revealed that the ticagrelor group demonstrated increased, yet not statistically significant, bone formation relative to the controls [70]. Overall, ticagrelor has yielded promising efficacy in the stimulation of bone regeneration, but additional studies on animal models are required before utilization in a clinical setting.

#### 3.3.3. ENT-1 Inhibitors

In addition to DIPY and ticagrelor, there have been other ENT-1 inhibitors studied to increase adenosine signaling and stimulate bone regeneration. The theory remains the same: to prevent adenosine transport across the cell membrane, causing the extracellular adenosine concentration to rise, thereby agonizing the respective adenosine receptors.

Deficiency of ENT-1 was first studied in vitro to understand the biochemical effects that its dysfunction would have on cellular response. Ii et al. harvested intravertebral discs (IVDs) from ENT-knockout mice and isolated the annulus fibrosus cells [76]. ENT-knockout cells demonstrated no adenosine uptake with increased alkaline phosphate activity and mineralization, which were further discovered in vivo [76]. Interestingly, ENT-knockout mice displayed ectopic bone formation in the paraspinal tissues of the cervical, thoracic, and lumbar spine, as well as calcification within IVDs [76]. The IVDs were found to have decreased expression of annulus fibrosus markers, indicating a disruption in cell differentiation [76]. Additionally, histology showed that alkaline phosphatase localized to the inner annulus fibrosus in ENT-knockout mice [76]. Another study on ENT-knockout mice by Hinton et al. analyzed the transporter’s effect on bone structure and mechanics with unexpected findings. Using X-ray, ENT-knockout mice were found to display decreased cervical and thoracic bone density compared to wild-type mice [75]. The knockout femurs also displayed diminished trabecular bone volume fraction, decreased trabecular thickness, and lower bone mineral density [75]. Further genetic expression analysis revealed increased osteoclast resorption activity in ENT-knockout mice [75]. These results contradict the findings of many of the aforementioned studies with regard to osteoclast activity. Mechanistically, it has been determined that ENT-1 inhibition increases extracellular adenosine, agonizing A_2A_ and A_2B_ and decreasing osteoclast differentiation and activity. Hinton et al.’s results claim otherwise.

In terms of translating the effect of ENT-1 inhibition into the clinical setting, it has been determined that this transporter can be inhibited by many marketed drugs, in addition to the ones previously mentioned. Nitrobenzylmercaptopurine ribose and nevirapine are notable inhibitors of ENT-1 activity, although their metabolism leaves them susceptible to many drug–drug interactions [108,109]. Jouan et al. studied the effects of 24 tyrosine kinase inhibitors on ENT-1 inhibition [109]. Five tyrosine kinase inhibitors were found to potently inhibit ENT-1 and, according to criteria provided by the United States Food and Drug Administration (US FDA), it was predicted that loratinib would have the potential for effective inhibition in vivo. Overall, ENT-1 inhibition is a clinically viable option to stimulate adenosine signaling; however, further mechanistic research is necessary to understand its full effect on bone regeneration.

#### 3.3.4. CD39/73 Agonist

Contrary to DIPY and ticagrelor, CD39 and CD73 agonists utilize a different mechanism of action to increase local adenosine activity. CD39 and CD73 are membrane-anchored enzymes responsible for dephosphorylating ATP, ADP, and AMP, all of which are necessary steps in the production of adenosine. By shifting these reactions towards adenosine formation, CD39 and CD73 agonists can stimulate adenosine signaling and subsequent bone regeneration.

Previous studies have described the mechanistic effects of CD73 agonism in vitro. Takedachi et al. studied primary osteoblasts harvested from calvaria of CD73-knockout mice [71]. These osteoblasts displayed significantly decreased alkaline phosphatase expression and delayed calcified nodule formation relative to CD73-expressing osteoblasts [71]. Additionally, CD73-expressing osteoblasts produced high levels of adenosine and were found to express A_2A_ and A_2B_, but not A_1_ or A_3_, as confirmed by Bertolini et al. [77]. However, because osteoclasts have lower CD73 expression, they produce less adenosine [77].

Contrary to many adenosine signaling studies emphasizing the effect of osteoblast stimulation via A_2A_, others determined that osteoblast stimulation occurred through the A_2B_ receptor [71]. This result was confirmed by Shih et al., finding that A_2B_ activation stimulates osteoblastogenesis and reduces osteoclastogenesis [73]. Shih et al. used a different approach to downregulate CD39 and CD73 activity [73]. By using ovariectomized mice, estradiol was lowered, emulating postmenopausal osteoporosis. CD39 and CD73 were downregulated in osteoblasts, osteoclasts, and macrophages, leading to decreased in extracellular adenosine. The direct A_2B_ agonist, BAY 60-6583, was shown to decrease osteoporosis and attenuate bone loss [73]. This study emphasized that other hormones and molecules affect the cellular response of osteoblasts and osteoclasts. Clinically, this poses a challenge and could impact the bone regeneration efficacy of these treatments in humans.

Due to the ability of CD73 to induce adenosine signaling, CD73-expressing mesenchymal stem cells have been tested as a therapy to stimulate bone regeneration [74]. These MSCs were found to have increased osteogenic potential relative to MSCs lacking CD73 expression [74]. When translating this to an in vivo mice model, administration of CD73-expressing MSCs was shown to increase bone callus formation and neovascularization, serving as a viable option to induce bone regeneration [74]. Similar findings from Takedachi et al. confirmed the utility of CD73 in bone regeneration, whereby CD73-deficient mice exhibited femoral osteopenia and diminished osteoblastic markers with decreased bone mineral content [71]. This result was confirmed yet again by another CD73 knockout mice model [72]. Bradaschia-Correa et al. demonstrated that CD73 knockout mice displayed delayed bone regeneration and reduced bone matrix deposition, once again highlighting the importance of CD73 in bone regeneration [72]. Despite these promising findings, overexpression of CD73 has been shown to induce neoplasm in other organs, necessitating caution in its systematic activation [110,111,112]. Overall, it is evident that CD73 is a necessary component of adenosine signaling and localized delivery shows promise in inducing adequate bone regeneration.

#### 3.3.5. Other Adenosine Metabolism Inhibitors

Another target in adenosine metabolism that has been studied in bone regeneration considers the degradation of adenosine. Adenosine deaminase is an enzyme that catalyzes the irreversible deamination of adenosine to form inosine [113]. Adenosine kinase is an enzyme that adds a phosphate group to adenosine, forming AMP [114]. By inhibiting these enzymes, one can theoretically elevate the extracellular concentration of adenosine, agonizing the respective adenosine receptors [57]. This mechanism has been studied mostly in the setting of arthritis, but some studies have noted its role in bone metabolism.

Sauer et al. studied the bone phenotype of adenosine deaminase-deficient mice. They found that these mice exhibited growth retardation with decreased trabecular density, which they attributed to an imbalance in the RANKL/osteoprotegerin axis [115]. In humans with adenosine deaminase deficiency, patients are found to have skeletal abnormalities, including scapular spurring and rib cupping, potentiating maladaptive effects on bone metabolism in the absence of adenosine deaminase [116,117]. On the contrary, a pre-clinical study by Tesch et al. found that increased adenosine due to adenosine deaminase and adenosine kinase inhibition created an anti-inflammatory environment that may prove to be therapeutic in arthritis pathogenesis [79]. While this mechanism has not been directly translated to bone regeneration, in theory, the increased adenosine from adenosine deaminase inhibition could agonize osteogenic adenosine pathways. Despite this, it is known that improper function of adenosine deaminase can cause a host of issues, as demonstrated by those with adenosine deaminase deficiency. These include severe immunodeficiency, neurodevelopmental effects, and pulmonary dysfunction [118]. As such, adenosine deaminase and adenosine kinase are less favorable targets to induce adenosine signaling for bone regeneration.

### 3.4. Future Directions

#### 3.4.1. Controlled Release of Adenosine for Bone Regeneration, Tendon Healing, and Reversal of Osteoarthritis

Adenosine’s half-life, lasting only seconds due to its deamination by adenosine deaminase or phosphorylation by adenosine kinase, severely limits its therapeutic utility. Yet, the clinical potential of adenosine-mediated osteogenesis has prompted the investigation of feasible delivery systems for its sustained and controlled release at defect sites. This would both prolong its osteogenic effect and reduce the undesirable side effects associated with systemic delivery. While only in the preliminary phases of exploration in vitro and in vivo, delivery of adenosine by core-shell nanofibers, microgels, nanoparticles, and liposomes has recently taken place. Potential applications of adenosine through such extended delivery systems include at sites of fracture, tendon tears, or osteoarthritic joints [61,119,120,121,122,123,124].

#### 3.4.2. Core–Shell Nanofibers

The use of coaxial electrospinning has been investigated as a plausible method of controlled release for adenosine. With this technology, adenosine may be loaded within a core–sheath structure composed of coaxial nanofibers [61]. Historically, this technology has been efficacious for the sustained delivery of osteogenic growth factors and antibiotics [125,126]. In a study performed by Cheng et al., the encapsulation of adenosine within co-axial poly(e-caprolactone) (PCL) and polyvinyl alcohol (PVA) nanofibers for targeted bone regeneration of full-thickness rat cranial defects were explored [61]. In comparison to sham and non-loaded PCL/PVA groups, adenosine-loaded PCL/PVA co-axial nanofibrous mats resulted in a significantly greater percentage of bone volume/total volume (BV/TV) at 4 and 8 weeks in vivo [61]. The effectiveness of this delivery system was largely attributed by the authors to the use of the synthetic PCL polymer. The hydrophobicity of PCL led to its slow degradation, allowing for the progressive release of adenosine from within the structure’s core in a linear pattern, which was even detectable at 60 days in vitro [61]. In addition, this study found that the core–shell nanofiber system of adenosine delivery did not result in the typical systemic effects associated with adenosine, such as hypotension, bradycardia, or hepatotoxicity [61].

#### 3.4.3. Microgels

In addition to core–shell nanofibers, microgel-based delivery of adenosine for bone healing is under active investigation. In a 2022 study conducted by Hoque et al., adenosine-loaded microgels were created via copolymerization of 3-acrylamidophenylboronic acid (3-APBA)- and 2-aminoethylmethacrylamide (2-AEMA)-conjugated hyaluronic acid (HA-AEMA) [120]. These were then utilized to generate an injectable 3D scaffold embedded with adenosine for application within a mouse tibia fracture site. By day 14, ~48% of the original adenosine content had been released from the scaffold in media. At 3 weeks in vivo, in comparison to non-adenosine loaded scaffolds, evidence of cortical bridging was only present in the adenosine-treated group, with a significantly higher BV/TV. The success found with this delivery system is attributed to the PBA moieties, which provided a boronate group for adenosine’s vicinal diol groups to load onto and to subsequently be released from [120].

#### 3.4.4. Nanoparticles and Liposomes

An additional method of adenosine delivery that has been recently explored is by amorphous calcium phosphate (ACP) nanoparticles, with ATP serving as the organic phosphorus source. A study performed by Liao et al. utilized ACP nanoparticles to augment healing at the site of an acute rotator cuff tear (RCT) in a rat model [119]. After 8 weeks in vivo and in comparison to the control and adenosine-only groups, a higher BV/TV ratio was detected within the ACP group, with more fibrocartilaginous regeneration seen after staining [119]. In addition, a series of in vitro studies found significantly enhanced tubule formation in the ACP group, also suggesting improved angiogenesis with its application [119]. It was postulated that the osteogenic and angiogenic effects of the ACP nanoparticles were partially attributable to their rich supply of adenosine as a result of ATP hydrolysis. These findings have important implications for the potential prevention of incomplete healing and re-tears often witnessed in RCT patients.

Attachment of adenosine to biodegradable nanoparticles has also been explored as a potential effective long-term therapy for osteoarthritis [122]. Cartilage homeostasis and regeneration have been noted to be maintained through stimulation of the A_2A_ receptor by adenosine [83,121]. Further, previous studies have shown that mice lacking A_2A_ receptors develop spontaneous osteoarthritis [121,124]. In this context, a recent study utilized copolymeric polyethylene glycol (PEG) and polylactic acid (PLA) nanoparticles bound to adenosine to prolong its therapeutic effect. Ultimately, intra-articular injection of the adenosine-functionalized PLA-PEG nanoparticles prevented the progression of post-traumatic osteoarthritis in a rat knee model [122]. Alternatively, liposomal delivery of adenosine is a recently developed method for prolonged release in the treatment of osteoarthritis. Several studies have found that intra-articular injection of liposomal suspensions of adenosine and a selective A_2A_ receptor agonist reversed osteoarthritis progression in both obesity-induced osteoarthritic mice and post-traumatic osteoarthritic rat models [123,124]. With future supporting preclinical studies, liposomal adenosine or adenosine-functionalized nanoparticles may serve as effective disease-modifying osteoarthritis therapies.

## 4. Discussion

The review of the preclinical status of indirect adenosine agonists for bone regeneration underscores the significant potential of adenosine signaling pathways in orthopedic therapeutics. Our examination of various compounds, including DIPY and ticagrelor, reveals a promising horizon for the indirect stimulation of adenosine receptors as a novel, non-surgical approach to bone healing and regeneration. The mechanisms of action identified through modulation of osteoblast activation and osteoclast differentiation highlight the intricate balance of bone homeostasis regulated by adenosine signaling.

While adenosine’s role in bone healing represents a significant and novel discovery, translating these findings into clinical applications remains a complex challenge. The preclinical evidence supporting adenosine-targeted therapy for bone regeneration is still being accumulated. However, several key factors need to be addressed to pave the way for clinical trials. Notably, the precise mechanisms by which adenosine promotes osteogenesis and modulates the bone microenvironment must be thoroughly understood. This includes detailed studies on the interactions between adenosine receptors and the cellular pathways involved in bone formation and healing. The diversity of adenosine receptor subtypes and their varying effects on different cell types add layers of complexity that must be further elucidated to develop targeted therapies. Furthermore, the development and optimization of localized delivery systems are critical. Controlled release technologies have shown promise in the preclinical models discussed, providing sustained adenosine release at the bone defect site, thereby enhancing bone regeneration while minimizing systemic side effects. Fine-tuning these delivery systems to ensure consistent and effective adenosine release tailored to the specific needs of different bone defects will be a significant step towards clinical application. Lastly, regulatory hurdles pose substantial challenges. Given that adenosine-based therapies are relatively novel, gaining approval for clinical trials from regulatory bodies like the US FDA involves rigorous safety and efficacy evaluations [127,128]. Regulatory agencies require comprehensive data from well-designed preclinical studies, which are largely underway, demonstrating not only the therapeutic benefits, but also the absence of adverse effects in the long term.

## 5. Conclusions

In synthesizing the findings from in vitro and in vivo studies, this review underscores the potential therapeutic value of adenosine signaling in bone regeneration. As we navigate the complexities of translating these preclinical insights into clinical realities, the goal remains to fulfill the unmet need for effective, non-surgical treatments for bone defects and fracture non-unions. The journey from the bench to the bedside, while challenging, is paved with the promise of novel biologics that may revolutionize the standard of care in craniomaxillofacial surgery, orthopedic surgery, and beyond.

## Figures and Tables

**Table 1:** In vivo studies examining the effects of indirect adenosine agonism on bone regeneration.

Drug	Target	Species	Location	Results	Reference
NA	A_2A_	Mice (n = 14)	Femur	A_2A_ knockout mice showed significantly lower ratios of bone volume to total volume with decreased bone mineral density and bone mineral content.	[52]
DIPY	ENT-1	Sheep (n = 15)	Vertebrae	The DIPY scaffold provided increased osteointegration three weeks postoperatively, while no differences were seen six or twelve weeks postoperatively.	[62]
DIPY	ENT-1	Pig (n = 14)	Calvarium	DIPY scaffolds increased bone growth relative to control, while maintaining native bone mechanics. No ectopic bone growth or excessive inflammation was seen.	[63]
DIPY	ENT-1	Rabbit (n = 18)	Alveolus	DIPY-coated scaffolds had statistically similar bone regeneration to rhBMP-2-coated scaffolds, while avoiding early suture fusion associated with rhBMP-2. There were no mechanical differences between bones.	[64]
DIPY	ENT-1	Rabbit (n = 16)	Calvarium/Alveolus	DIPY scaffolds promoted osteogenic growth better than autologous bone grafts, with mechanical properties, vascularization, and organization comparable to native bone. DIPY scaffolds did not result in premature closure of craniofacial sutures or disruption of facial symmetry.	[65]
DIPY	ENT-1	Mice (n = 120)	Calvarium	DIPY enhanced bone regeneration with increased osteoblasts and decreased osteoclasts relative to controls. DIPY showed accelerated bone regeneration relative to BMP-2 treatment. A_2A_ knockout mice did not show enhanced bone regeneration with DIPY.	[17]
DIPY	ENT-1	Rabbit (n = 15)	Mandible	The DIPY treatment group demonstrated the most bone regeneration relative to control and collagen groups with no inflammatory response.	[66]
DIPY	ENT-1	Rabbit (n = 16)	Calvarium	DIPY-treated scaffolds demonstrated significantly more bone growth than control groups. No ectopic bone formation was noted in the DIPY-treated group.	[67]
DIPY	ENT-1	Rabbit (n = 24)	Radius	3D-printed scaffolds treated with DIPY showed increased bone regeneration in a dose-dependent manner, while maintaining the biomechanical properties of native bone.	[68]
Ticagrelor	ENT-1	Mice (n = 120)	Calvarium	Increased bone area, bone volume, and bone mineral density with an increase in alkaline phosphatase expression.	[69]
Ticagrelor	ENT-1	Rats (n = 40)	Tibia	Higher, but not statistically significant, new bone formation relative to implants treated with acetylsalicylic icracid, clopidogrel, or prasugrel.	[70]
NA	CD73	Mice	Femur/Tibia	CD73 knockout mice had lower bone mineral density, reduced trabecular bone volume, and decreased trabecular thickness. They exhibited decreased osteoblast differentiation.	[71]
NA	CD73	Mice (n = 20)	Tibia	CD73 knockout mice exhibited delayed bone regeneration and decreased bone matrix deposition with decreased callus size following fracture. These mice had reduced cell proliferation, alkaline phosphatase activity, and osteoclast numbers, suggesting that CD73 may be necessary to induce osteoblast activity and stimulate bone regeneration.	[72]
BAY 60-6583	A_2B_	Mice (n = 12)	Vertebrae	Osteoporotic animals (secondary to low estradiol) exhibit decreased CD73 and CD39 with lower extracellular adenosine. An A_2B_ agonist, BAY 60-6583, was administered and showed decreased vertebral and femoral bone loss with increased bone density despite no changes in CD73/39 expression.	[73]
CD73(+) mesenchymal stem cells (MSCs)	CD73	Mice (n = 15)	Femur	MSCs expressing CD73 contributed to the endochondral ossification process immediately after fracture and continued to differentiate into osteoblasts and subsequently osteocytes throughout the fracture healing process. CD73+ cells were also found to contribute to neovascularization at the fracture site. CD73+ MSCs were grafted at the fracture site, showing increased bone callus and lamellar bone formation.	[74]
NA	ENT-1	Mice (n = 18)	NA	ENT-1 knockout mice showed reduced bone density and bone mineral density relative to controls in the thoracic and lumbar spine and femur. ENT-1 knockout mice were also found to have increased TRAP expression in long bones.	[75]
NA	ENT-1	Mice (n = 16)	Vertebrae	ENT-1 knockout mice showed increased calcification and hypermineralization of intravertebral discs.	[76]

**Table 2:** In vitro studies examining the effects of indirect adenosine agonism on bone regeneration.

Target	Drug	Cell Line	Location	Results	Reference
ENT-1	Ticagrelor	Bone marrow cells	Femur/Tibia	Inhibited osteoclastogenesis in an adenosine concentration-dependent manner with decreased receptor activator of NF-κB ligand (RANKL) expression and increased osteoprotegerin expression.	[69]
CD73	NA	Osteoblasts	Calvarium	CD73-deficient osteoblasts exhibited decreased alkaline phosphatase expression with delayed calcification. Over-expression of CD73 led to accelerated osteoblast differentiation and increased adenosine receptor expression, which was stimulated by A_2B_ signaling.	[71]
CD73/39	NA	Bone marrow cells	Femur/Tibia/Humerus/Radius/Ulna/Vertebra	Bone marrow cells from osteoporotic bones (estradiol deficient) showed decreased expression of CD73 and CD39 with a significant decrease in extracellular adenosine. Increased extracellular adenosine and A_2B_ signaling were shown to promote osteoblastogenesis, decrease osteoclast differentiation, and reduce osteoclast transcription factor and TRAP.	[73]
CD73	Anti-CD73 monoclonal antibody (moAb)/adenosine 5′-(α,β-methylene) diphosphate (APCP)	Cancer stem cells/Osteoblasts/Osteoclasts	NA	Higher levels of CD73 were discovered in osteoblasts versus osteoclasts, leading to higher adenosine production by osteoblasts. Osteoclasts primarily expressed A_3_, while osteoclasts and osteoblasts expressed A_1_ and A_2A_. Osteoblasts expressed A2B. Both anti-CD73 moAb and APCP blocked CD73, decreasing adenosine concentrations.	[77]
A_1_	Rolofylline	Bone marrow cells	Femur/Tibia	Rolofylline blocked A_1_ and suppressed osteoclast differentiation, demonstrated by decreased expression of osteoclast-specific genes. This effect remained even in CD39 and CD73 deficient mice, indicating that A_1_ is the primary component for osteoclast differentiation.	[78]
CD73	CD73 MSCs	Bone marrow cells	Femur/Tibia	CD73+ MSCs showed increased plasticity and osteogenic potential relative to the CD73- counterparts.	[74]
ENT-1	CGS21680/DIPY	Osteoclasts	Femur/Tibia	Activation of A_2A_ by CGS21680 inhibited osteoclastogenesis in a dose-dependent manner. A_2A_ agonism decreased bone resorption and resulted in decreased concentrations of IL-1B.	[52]
ENT-1	CGS21680/DIPY	Osteoblasts/Osteoclasts	Calvarium	CGS21680 and DIPY inhibited osteoclasts, decreasing cathepsin K and RANK, while activating osteoblasts, showing increased levels of osteocalcin and osteonectin expression.	[17]
Adenosine Deaminase	Erythro-9-(2-hydroxy-3-nonyl) adenine hydrochloride (EHNA)	Chondrocytes	MTP/MCP Joints	Administration of EHNA did not significantly increase the extracellular concentration of adenosine. However, EHNA in combination with 5′-iodotubercidin (ITU) demonstrated a synergistic effect and largely increased concentrations of adenosine.	[79]
Adenosine Kinase	ITU	Chondrocytes	MTP/MCP Joints	ITU administration demonstrated significant increases in extracellular adenosine in a time-dependent manner.	[79]
ENT-1	NA	Annulus Fibrosus Cells	Intravertebral Discs	ENT-1 knockout cells from the annulus fibrosus displayed hyper mineralization and increased alkaline phosphatase activity, resulting in calcifications.	[76]
ENT-1	Ticagrelor	MSCs	Adipose Tissue	MSCs in the ticagrelor group demonstrated increased extracellular matrix formation, increased alkaline phosphatase activity, and decreased IL-6 and TNF-α expression.	[80]
